# Effects of the Structure of Benzenesulfonate-Based Draw Solutes on the Forward Osmosis Process

**DOI:** 10.3390/membranes12111067

**Published:** 2022-10-29

**Authors:** DaEun Yang, Yeonsu Cho, Hyo Kang

**Affiliations:** BK-21 Four Graduate Program, Department of Chemical Engineering, Dong-A University, 37 Nakdong-daero 550 Beon-gil, Saha-gu, Busan 49315, Korea

**Keywords:** forward osmosis, thermoresponsive, ionic liquid, draw solute, lower critical solution temperature, benzenesulfonate derivative

## Abstract

A series of phosphonium-based ionic liquids (ILs) based on benzenesulfonate derivatives (tetrabutylphosphonium benzenesulfonate ([TBP][BS]), tetrabutylphosphonium 4-methylbenzenesulfonate ([TBP][MBS]), tetrabutylphosphonium 2,4-dimethylbenzenesulfonate ([TBP][DMBS]), and tetrabutylphosphonium 2,4,6-trimethylbenzenesulfonate ([TBP][TMBS])) were synthesized via anion exchange with tetrabutylphosphonium bromide ([TBP][Br]). Then, we characterized the ILs and investigated their suitability as draw solutes for forward osmosis (FO), focusing on their thermoresponsive properties, conductivities, and osmotic pressures. We found that aqueous [TBP][BS] was not thermoresponsive, but 20 wt% aqueous [TBP][MBS], [TBP][DMBS], and [TBP][TMBS] had lower critical solution temperatures (LCSTs) of approximately 41, 25, and 21 °C, respectively, enabling their easy recovery using waste heat. Based on these findings, 20 wt% aqueous [TBP][DMBS] was tested for its FO performance, and the water and reverse solute fluxes were found to be approximately 9.29 LMH and 1.37 gMH, respectively, in the active layer facing the draw solution (AL-DS) mode and 4.64 LMH and 0.37 gMH, respectively, in the active layer facing the feed solution (AL-FS) mode. Thus, these tetrabutylphosphonium benzenesulfonate-based LCST-type ILs are suitable for drawing solutes for FO process.

## 1. Introduction

The scarcity of clean water and water pollution are two serious global environmental problems that have emerged in recent decades as a result of continued industrial growth and agricultural activities [1,2,3]. To solve these environmental issues, seawater desalination and wastewater recycling are considered good solutions [4,5].

Forward osmosis (FO) is a desalination membrane technology that drives water flow from the feed to permeate the side of a membrane across an osmotic pressure gradient. To achieve the permeation of water, a membrane is placed between the feed and draw solutions, and this creates a high osmotic pressure (higher concentration compared to the feed solution). The FO process has received much attention owing to its advantages, including high water recovery, high energy efficiency, low susceptibility to membrane contamination, and simple operation [6,7,8]. FO is generally an energy-efficient process; however, more research into how to regenerate the draw solute from the diluted draw solution for reuse and to produce purified water is required. In particular, achieving a balance between high osmotic pressure and easy regeneration is a goal but is difficult to achieve in practice [9].

There are two main types of draw solutions: nonresponsive and responsive draw solutes. Nonresponsive draw solutes do not respond to external stimuli, such as pH changes, light, temperature, or electromagnetic fields, and show no significant change in their water affinity after stimulation. To date, inorganic salts, such as NaCl, (NH_4_)_2_SO_4_, MgCl_2_, Na_2_SO_4_, KHCO_3_, and Ca(NO_3_)_2_, have been studied as draw solutes [10,11,12]. In addition, organic compounds have also been used as nonresponsive draw solutes [13,14], and studies into the use of nonresponsive draw solutes have mainly focused on the enhancement of osmotic pressure and reduction of reverse diffusion [15]. Responsive draw solutes, in contrast, are considered to be smart draw solutes because they can switch from hydrophilic to hydrophobic in response to external stimuli, such as temperature, magnetic or electric fields, pH changes, or light. Therefore, with appropriate responsive draw solutes, regeneration can be easily and efficiently achieved while maintaining a sufficiently high drawing ability. In particular, thermoresponsive draw solutes are of interest because of their unique ability to become miscible or immiscible in water depending on the temperature. These draw solutes are cost-effective and can exploit green energy, such as low-grade industrial waste heat and solar energy. Many compounds have been suggested as thermally responsive draw solutes, including magnetic nanoparticles, polymers, hydrogels, and ionic liquids (ILs) [16,17,18,19].

Functionalized magnetic nanoparticles could be used as a draw solute system. For example, functionalized magnetic nanoparticles with poly(sodium styrene-4-sulfonate)-*co*-poly(*N*-isopropylacrylamide) (PSSS-PNIPAM) and triethylene glycol have been investigated as thermoresponsive draw solutes [20,21]. For these thermoresponsive magnetic nanoparticles, it is known that factors, such as particle size, the distribution of hydrophilic groups, and the number of hydrophilic groups, are closely related to FO performance [22]. Very recently, thermoresponsive nonionic amphiphilic copolymers using poly(ethylene oxide)-*block*-poly(propylene oxide)-*block*-poly(ethylene oxide) (PEO-PPO-PEO) copolymers as the draw solute have been reported [23]. The thermoresponsive behavior could be controlled by changing the composition of the copolymers. In addition to the (PEO-PPO-PEO) copolymers, various polymers, such as poly(*N*-*n*-propylacrylamide) (PNPAm), methyl cellulose, glycerol-oligo(ethylene oxide)_m_-*block*-oligo(butylene oxide)_n_ (GE_m_B_n_), and poly(vinyl caprolactone), have been known to display thermoresponsive behaviors [24,25,26,27]. Various thermally responsive hydrogels were also studied as draw solutes for the FO process, such as poly(*N*-isopropylacrylamide)-*co*-sodium acrylate (P(NIPAM-*co*-SA)), poly(acrylamide), poly(*N*-isopropylacrylamide), and poly(*N*-isopropylacrylamide)-*co*-deep eutectic mixture (P(NIPAM-*co*-DEM)) hydrogels [28,29,30]. Although hydrogels are widely studied as draw solutes because they have a low reverse solute flux in the FO process, there are still some issues to be solved, including low water flux, high external concentration polarization, and in-continuous operation [31,32]. Recently, responsive ILs have been proven to have significant potential as draw agents owing to their high ionicity, high thermal stability, and negligible vapor pressure [33,34]. ILs are salts that can be tailored to have thermally responsive properties by balancing the cationic and anions components [35]. Most ILs are liquids at room temperature. In general, the cations include alkylphosphonium, alkylammonium, alkylsulfonium, *N,N*′-dialkylimidazolium, and alkylpyridinium moieties [36,37,38,39], whereas the anions include large organic anions, such as tetracyanoborate, carboxylate, tetrafluoroborate, and bis-(trifluoromethanesulfonyl) amide [40,41,42,43]. Recently, thermoresponsive ILs have been used to draw solutes in FO [44]. Crucially, the structural diversity of ILs is so wide that their physicochemical properties, such as polarity, melting point, hydrophobicity, and viscosity, can be controlled by altering the structure of the constituent ions [45,46,47]. Further, the ionic strength and high-water solubility of ILs suggest that they could generate sufficient osmotic driving force to induce water permeation across a membrane [48,49].

Thermoresponsive ILs show a dramatic change in water solubility in response to temperature changes and can be divided into two types: upper critical solution temperature (UCST) and/or lower critical solution temperature (LCST) types. In the UCST type, the ILs are soluble in water, owing to the hydrogen-bonding interactions between the IL and water molecules at high temperatures. Upon cooling below the cloud point temperature (*T_cp_*), the hydrogen bonds break, and hydrophobic interactions become dominant, turning the IL insoluble and the solution turbid. For the LCST-type, at low temperatures, the ILs are soluble in water because of the hydrogen-bonding interactions between IL/water molecules. On heating above the *T_cp_*, the hydrogen bonds break, and hydrophobic interactions become dominant, making the ILs insoluble and the solution turbid. The advantage of thermoresponsive IL draw solutes, especially the LCST type, is that the hydrated thermoresponsive IL draw solute in the diluted draw solution can be separated from water by inducing a mild temperature change, and most of the draw solute or water can be easily recovered through liquid–liquid phase separation [50,51]. Therefore, FO processes using thermoresponsive draw solutes have lower regeneration energy requirements than other draw solutes because the thermal energy can be obtained from geothermal or low-grade industrial waste heat [52,53,54,55].

In this study, we synthesized several types of tetrabutylphosphonium-based ILs having different anions, including benzenesulfonate (BS), 4-methylbenzenesulfonate (MBS), 2,4-dimethylbenzenesulfonate (DMBS), and 2,4,6-trimethylbenzenesulfonate (TMBS). Further, we conducted systematic analyses of their thermoresponsive phase-separation behavior and osmolality generation characteristics. We also investigated the potential of an aqueous solution of the tetrabutylphosphonium benzenesulfonate-based IL as the draw solute for the FO process.

## 2. Materials and Methods

### 2.1. Materials

Sodium benzenesulfonate (>96%), sodium 4-methylbenzenesulfonate, sodium 2,4-dimethylbenzenesulfonate (>98%) sodium 2,4,6-trimethylbenzensulfonate (>98%), and tetrabutylphosphonium bromide (99%) were obtained from Tokyo Chemical Industry Co., Ltd. (TCI), (Tokyo, Japan) and used without purification. Dichloromethane and deionized (DI) water (for total organic carbon (TOC) analysis) were purchased from Sigma-Aldrich Co., LLC. (Saint Louis, MO, USA), and used as received. All other chemicals were purchased without further purification or processing.

### 2.2. Synthesis of LCST-Type ILs

Four thermally responsive draw solutes were investigated in this study. [TBP][BS] was prepared by dissolving [TBP][Br] (4.00 g, 10 mmol) and sodium benzenesulfonate ([Na][BS]) (3.60 g, 20 mmol) in DI water (28 mL) in a flask. After dissolution, the mixture was stirred at room temperature for 24 h. The product was extracted with dichloromethane (80 mL, three times), washed three times with DI water (40 mL), and dried over anhydrous magnesium sulfate. The solvent was removed using a rotary evaporator to yield a product. ^1^H−NMR [400 MHz, D_2_O, δ/ppm]: 0.75−0.98 (t, 12H, P^+^-(CH_2_-CH_2_-CH_2_-*CH*_3_)_4_), 1.23−1.62 (m, 16H, P^+^-(CH_2_-*CH*_2_*-CH*_2_-CH_3_)_4_)), 2.10−2.23 (m, 8H, P^+^-(*CH*_2_-CH_2_-CH_2_-CH_3_)_4_), 7.52−7.75 (s, 3H, *PhH*-SO_3_^−^), and 7.75−7.83 (s, 2H, *PhH*-SO_3_^−^). HRMS (ESI, positive mode); *m*/*z*: Found 259.2558; calculated for C_16_H_36_P, 259.2555. HRMS (ESI, negative mode); *m*/*z*: Found 156.9961; calculated for C_6_H_5_O_3_S, 156.9959.

[TBP][MBS] was prepared by dissolving [TBP][Br] (6.80 g, 20 mmol) and [Na][MBS] (7.77 g, 40 mmol) in DI water (28 mL) in a flask. After dissolution, the mixture was stirred at room temperature for 24 h. The product was then extracted with dichloromethane (80 mL, three times), washed three times with DI water (40 mL), and dried over anhydrous magnesium sulfate. The solvent was removed using a rotary evaporator to obtain a product. ^1^H−NMR [400 MHz, CDCl_3_, δ/ppm]: 0.83−1.06 (t, 12H, P^+^-(CH_2_-CH_2_-CH_2_-*CH*_3_)_4_), 1.38−1.62 (m, 16H, P^+^-(CH_2_-*CH*_2_*-CH*_2_-CH_3_)_4_), 2.17−2.42 (m, 8H, P^+^-(*CH*_2_-CH_2_-CH_2_-CH_3_)_4_, s, 3H, *CH*_3_-Ph-SO_3_^−^), 7.00−7.14 (s, 2H, CH_3_-*PhH*-SO_3_^−^), and 7.71−7.83 (s, 2H, CH_3_-*PhH*-SO_3_^−^). HRMS (ESI, positive mode); *m*/*z*: Found 259.2552; calculated for C_16_H_36_P, 259.2555. HRMS (ESI, negative mode); *m*/*z*: Found 171.0119; calculated for C_7_H_7_O_3_S, 171.0116.

[TBP][DMBS] was prepared by dissolving [TBP][Br] (6.80 g, 20 mmol) and [Na][DMBS] (8.33 g, 40 mmol) in DI water (28 mL) in a flask. After dissolution, the mixture was stirred at room temperature for 24 h. The product was then extracted with dichloromethane (80 mL, three times), washed three times with DI water (40 mL), and dried over anhydrous magnesium sulfate. The solvent was removed using a rotary evaporator to obtain a product. ^1^H−NMR [400 MHz, CDCl_3_, δ/ppm]: 0.79−1.03 (t, 12H, P^+^-(CH_2_-CH_2_-CH_2_-*CH*_3_)_4_), 1.35−1.63 (m, 16H, P^+^-(CH_2_-*CH*_2_*-CH*_2_-CH_3_)_4_), 2.20−2.27 (s, 3H, *CH*_3_-Ph-SO_3_^−^), 2.28−2.37 (m, 8H, P^+^-(*CH*_2_-CH_2_-CH_2_-CH_3_)_4_), 2.62−2.74 (s, 3H, *CH*_3_-Ph-SO_3_^−^), 6.86−6.92 (d, 1H, CH_3_-*PhH*-SO_3_^−^), 6.93−6.96 (s, 1H, CH_3_-*PhH*-SO_3_^−^), and 7.78−7.91 (d, 1H, CH_3_-*PhH*-SO_3_^−^). HRMS (ESI, positive mode); *m*/*z*: Found 259.2553; calculated for C_16_H_36_P, 259.2555. HRMS (ESI, negative mode); *m*/*z*: Found 185.0273; calculated for C_8_H_9_O_3_S, 185.0272.

[TBP][TMBS] was prepared by dissolving [TBP][Br] (6.80 g, 20 mmol) and [Na][TMBS] (8.89 g, 40 mmol) in DI water (28 mL) in a 250 mL flask. After dissolution, the mixture was stirred at room temperature for 24 h. The product was extracted with dichloromethane (80 mL, three times), washed three times with DI water (40 mL), and dried over anhydrous magnesium sulfate. The solution was removed using a rotary evaporator to give an oily liquid product. ^1^H−NMR [TBP][TMBS] [400 MHz, CDCl_3_, δ/ppm]: 0.78−1.04 (t, 12H, P^+^-(CH_2_-CH_2_-CH_2_-*CH*_3_)_4_), 1.38−1.58 (m, 16H, P^+^-(CH_2_-*CH*_2_*-CH*_2_-CH_3_)_4_), 2.11−2.23 (s, 3H, *CH*_3_-Ph-SO_3_^−^), 2.28−2.42 (m, 8H, P^+^-(*CH*_2_-CH_2_-CH_2_-CH_3_)_4_), 2.62−2.75 (s, 6H, *CH*_3_-Ph-SO_3_^−^), and 6.67−6.84 (d, 2H, CH_3_-*PhH*-SO_3_^−^). HRMS (ESI, positive mode); *m*/*z*: Found 259.2553; calculated for C_16_H_36_P, 259.2555. HRMS (ESI, negative mode); *m*/*z*: Found 199.0432; calculated for C_9_H_11_O_3_S, 199.0429.

### 2.3. Characterization

^1^H−NMR spectroscopy (MR400 DD2 NMR spectrometer, Agilent Technologies, Inc. (Santa Clara, CA, USA), Fourier transform infrared (FT−IR) spectrometry (NICOLET iS20, Thermo Fisher Scientific Inc., Waltham, MA, USA), and a high−resolution mass spectrometer (HRMS, maXis HD, Bruker Corp., Billerica, MA, USA) were used to confirm the structures of the synthesized ILs. After preparing each draw solution, the conductivity (Mettler Toledo Seven2Go Pro, Zurich, Switzerland) was measured, and the osmotic pressure was determined by measuring the freezing point of the sample using an osmometer (K-7400, Knauer Co., Berlin, Germany). The LCST was determined by measuring the transmittance of aqueous solutions at 650 nm using an ultraviolet–visible (UV–Vis) spectrophotometer (EMC-11D-V, EMCLAB Instruments GmbH Co., Duisburg, Germany). The water flux was determined by comparing the height difference of the draw solution in the tube during FO operation. The reverse solute flux was measured using a conductivity meter by comparing the difference in conductivity before and after the FO test.

### 2.4. FO Tests

The flux was measured using a laboratory-scale FO system connected to a glass tube (L-shaped) [56]. A thin-film porous FO membrane (Hydration Technologies Inc. (Albany, OR, USA), HTI-TFC) was placed between the two glass tubes. One L-shaped tube was filled with DI water, whereas the other contained the IL solution as the draw solution. During the tests, stirring was continued with a magnetic stir bar and the temperature was maintained at room temperature. The water permeation flux was calculated from the volume of the draw solution before and after the FO test, as shown in Equation (1).

(1) $$J_{V}=\frac{\Delta V}{A\Delta t}$$

here, *J_V_* is the water permeation flux in the FO process (L m^−2^ h^−1^ or LMH), Δ*V* (L) is the volume change of the draw solution over time Δ*t* (h), and *A* (m^2^) is the surface area of the FO membrane, which was calculated to be 3325 × 10^−4^ m^2^.

The reverse solute flux (*J_s_*) was determined from the amount of draw solute that diffused into the feed solution and was calculated from the TOC value of the feed solution. The reverse solute flux in the FO process (g m^−2^ h^−1^ or gMH) was calculated from the difference in the conductivity of the feed solution before and after the FO test using Equation (2).

(2) $$J_{S}=\frac{\Delta \left(CV\right)}{A\Delta t}$$

here, Δ*C* (mol L^−1^) is the concentration of change in the feed solution after time Δ*t*, and Δ*V* (L) is the volume change after time Δ*t*.

## 3. Results and discussion

### 3.1. Synthesis and Characterization of LCST-Type ILs

**Figure 1:**
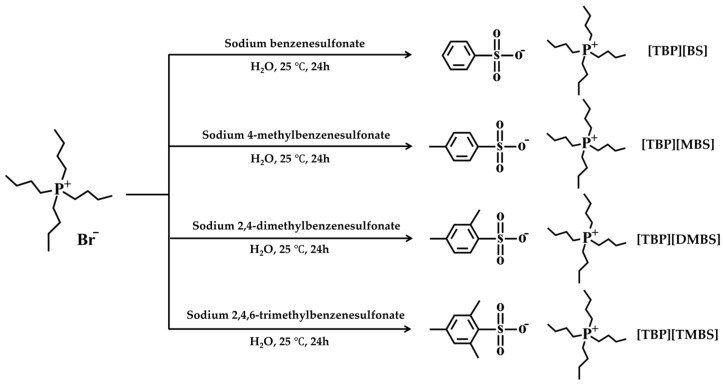
Synthetic route of [TBP][BS], [TBP][MBS], [TBP][DMBS], and [TBP][TMBS].

Figure 1 shows the chemical structures of the LCST-type ILs ([TBP][BS], [TBP][MBS], [TBP][DMBS], and [TBP][TMBS]) prepared by the ion-exchange reaction of tetrabutylphosphonium bromide and the sodium benzenesulfonate series.

**Figure 2:**
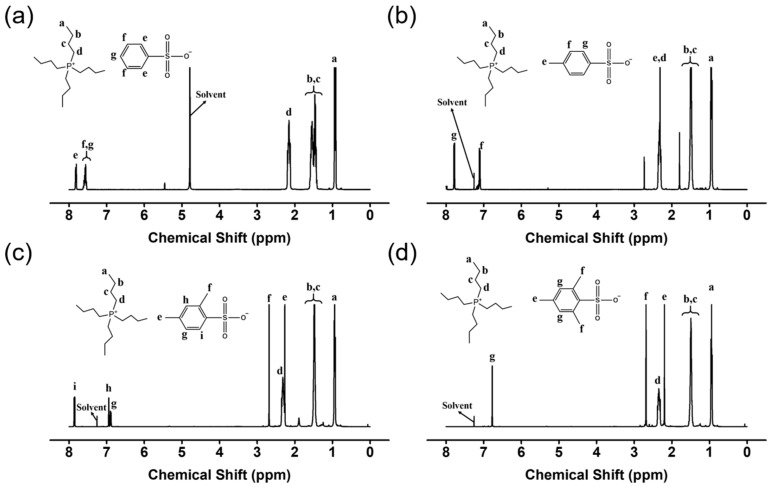
Proton nuclear magnetic resonance (^1^H−NMR) spectrum of (**a**) [TBP][BS], (**b**) [TBP][MBS], (**c**) [TBP][DMBS], and (**d**) [TBP][TMBS].

The chemical structure of the IL was analyzed by ^1^H−NMR and FT−IR spectroscopy and HRMS before the FO experiment. The ^1^H−NMR spectra and assignments are shown in Figure 2. The spectrum of [TBP][BS] contains peaks corresponding to protons in the phenyl ring of the benzenesulfonate moiety (δ = 7.52–7.75 (peaks g and f), δ = 7.75–7.83 ppm (peak e)) and those in the alkyl groups of the tetrabutylphosphonium moiety (δ = 0.75–0.98 (peak a), δ = 1.23–1.62 (peaks b and c), and δ = 2.10–2.23 ppm (peak d)), which suggests the inclusion of tetrabutylphosphonium moieties in [TBP][BS]. The ^1^H−NMR spectra of [TBP][MBS], [TBP][DMBS], and [TBP][TMBS] contain peaks corresponding to a larger number of alkyl protons than those in the spectrum of [TBP][BS]: δ = 2.17–2.42 (peak e, Figure 2b), δ = 2.20–2.27, δ = 2.62–2.74 (peaks e and f, Figure 2c), and δ = 2.11–2.23, δ = 2.62–2.75 (peaks e and f, Figure 2d), respectively. Thus, the ^1^H−NMR spectra confirm the successful production of ILs having different numbers of methyl groups in the anions.

**Figure 3:**
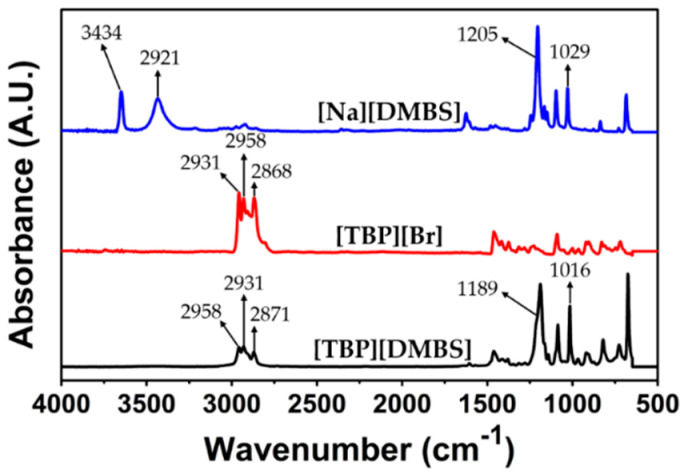
Fourier transform infrared (FT−IR) spectra of [Na][DMBS], [TBP][Br], and [TBP][DMBS] at room temperature.

**Figure 4:**
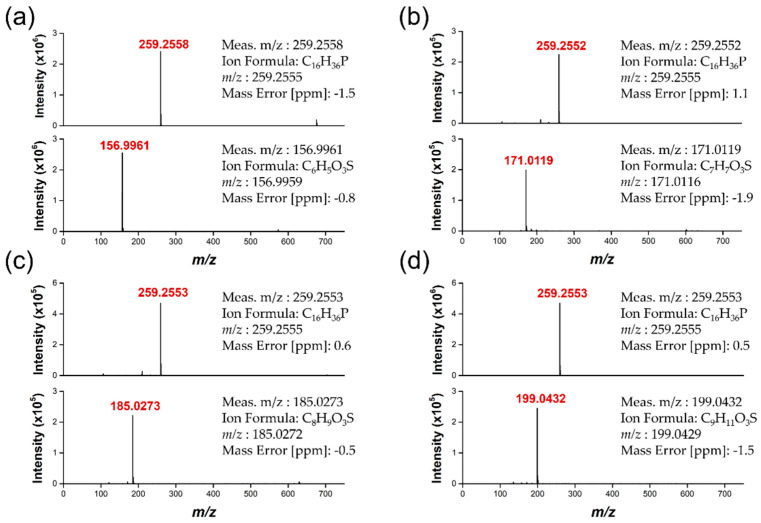
High−resolution mass spectrometer (HRMS) spectra of (**a**) [TBP][BS], (**b**) [TBP][MBS], (**c**) [TBP][DMBS], and (**d**) [TBP][TMBS].

In addition, LCST-type ILs confirmed anion exchange from [TBP][Br] to [TBP][DMBS] using FT−IR spectroscopy as shown in Figure 3. Fortunately, [Na][DMBS] has no IR absorbance in the saturated C-H region, indicating that the absorption of [TBP][DMBS] from 2960–2870 cm^−1^ corresponds to the butyl groups in [TBP][Br]. Therefore, this region can be used to discuss changes in the cations. The absorption at 1600–1500 cm^−1^ can be assigned to the stretching of the C=C bond in the benzene ring, but the coarse spectrum makes it difficult to track changes in this region [57]. On the other hand, the sulfonate group appeared as bands generated from the asymmetric stretching vibrations of SO_3_^−^ at 1350–1200 cm^−1^. However, the peaks located at 1100–1010 cm^−1^ correspond to the symmetric stretching of SO_3_^−^, which makes it possible to follow changes in the anions. The FT−IR peaks of the measured sulfonate groups were observed at wavenumbers similar to those previously reported [58,59]. Consequently, the bands corresponding to the C-H and S=O groups were useful for the interpretation of the IL structures. The molecular weight of ILs was measured by HRMS, data showed in Figure 4, the actual measured molecular weight of both the cation and anion portion of the ILs was found almost consistent with the calculated value, respectively.

### 3.2. Conductivity

**Figure 5:**
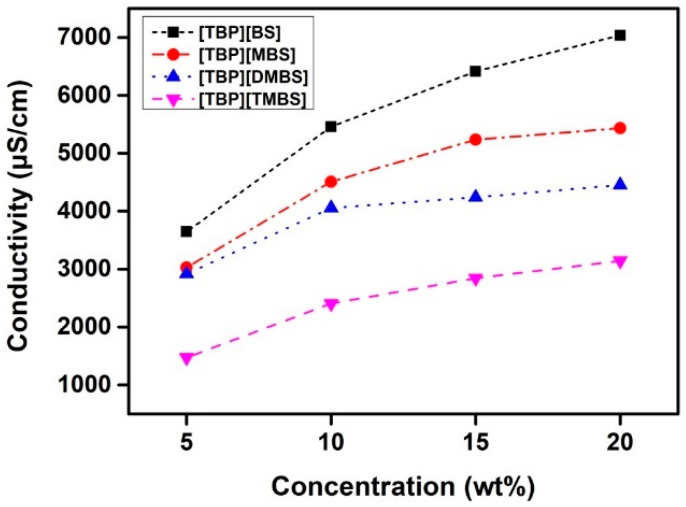
Conductivity of [TBP][BS], [TBP][MBS], [TBP][DMBS], and [TBP][TMBS] aqueous solutions according to solution concentration.

Conductivity is an indicator of the number of solute ions in the draw solution, as well as the degree of ion mobility. Furthermore, conductivity is affected by the degree of dissociation of ions, which is related to the osmotic pressure. In general, more conductive draw solute ions result in higher osmotic pressures [60]. Figure 5 shows the conductivities of aqueous solutions of the ILs at concentrations of 20, 15, 10, and 5 wt% at room temperatures. The conductivities of [TBP][BS], [TBP][MBS], [TBP][DMBS], and [TBP][TMBS] were approximately 5460, 4508, 4055, and 2406 μS cm^−1^, respectively, at 10 wt% and approximately 7037, 5436, 4452, and 3143 μS cm^−1^, respectively, at 20 wt%. Thus, the conductivity of the ILs is proportional to their concentration, as reported previously [61,62,63]. This result indicates that the ILs dissociate well in water, resulting in a high ion concentration. The conductivities of the IL draw solutions decreased in the order [TBP][BS] > [TBP][MBS] > [TBP][DMBS] > [TBP][TMBS] at all solution concentrations tested, and this indicates that [TBP][BS] should produce a higher osmotic pressure than [TBP][MBS], [TBP][DMBS], and [TBP][TMBS]. Correspondingly, the FO performance of [TBP][BS] as the draw solution was better than those of the other ILs, as discussed later. This seems to be due to the structural differences in the substituent groups of each IL. Interestingly, the conductivities of the aqueous IL solutions at all solution concentrations decreased with an increase in the number of methyl substituents on the benzene ring. The main factors affecting the conductivity of ILs are ion mobility and the volume of the functional groups [64,65]. In particular, increasing the number of methyl groups in the benzene ring increases ion aggregation and decreases ion mobility, leading to decreased ionic conductivity [66]. The decrease in conductivity can also be explained by the fact that, when a methyl group is attached to the benzene ring, the torsion angle between the conjugated rings increases, which increases the steric strain as the distance between the IL units increases [67,68]. Therefore, as the number of substituents in the aromatic ring increases, the ion mobility decreases and the torsion angle increases compared to the single substituted derivative, resulting in a decrease in the electrical conductivity.

### 3.3. Osmotic Pressure

In the FO process, osmosis drives the spontaneous diffusion of water from a feed solution having a lower osmotic potential to a draw solution having a higher osmotic potential. Therefore, the draw solute must have high diffusion in aqueous solution to achieve the active diffusion of water molecules from the influent solution towards the draw solution. The osmotic pressure is a function of solution concentration and is an indicator of FO performance and can be described by the Van ’t Hoff equation (Equation (3)).

*Π* = *C_i_RT* (3)

here, *Π* is the osmotic pressure, *C_i_* is the molar concentration of solute *i* in the dilute solution, *R* is the gas constant, and *T* is the absolute temperature.

**Figure 6:**
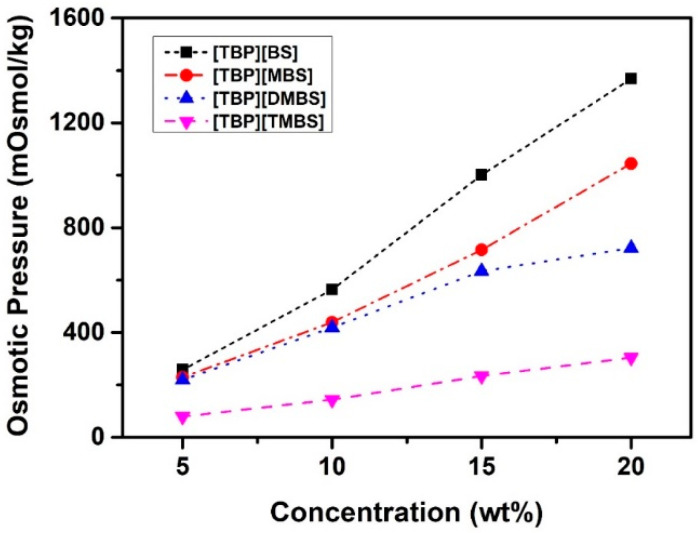
Osmotic pressure according to the concentration of [TBP][BS], [TBP][MBS], [TBP][DMBS], and [TBP][TMBS] measured by the freezing point depression method.

The osmotic pressure was measured using the freezing point depression method to study the possible applications of the ILs as draw solutes because the difference in osmotic pressure between the draw and feed solutions is the driving force behind the FO process. The osmotic pressure of the ILs as a function of their concentration in water is shown in Figure 6. The osmotic pressures of [TBP][BS], [TBP][MBS], [TBP][DMBS], and [TBP][TMBS] were approximately 564, 439, 419, and 144 mOsmol kg^−1^, respectively, at 10 wt%, increasing to approximately 1368, 1045, 722, and 304 mOsmol kg^−1^, respectively, when the concentration increased to 20 wt%. This result proves the Van ’t Hoff equation (Equation (3)). Additionally, the osmotic pressure decreased from [TBP][BS] to [TBP][TMBS] at all concentration ranges because of the known colligative property that depends on the concentration of the solute (ionic species or neutral molecules) [69]. Specifically, although the weight concentration is the same, the molecular weights of [TBP][MBS], [TBP][DMBS], and [TBP][TMBS] are greater than those of [TBP][BS]; therefore, the molar concentration of [TBP][BS] is higher than those of [TBP][MBS], [TBP][DMBS], and [TBP][TMBS], and the osmotic pressure of [TBP][BS] is higher. In addition, according to Van ’t Hoff equation, the osmotic pressure is correlated with the Van ’t Hoff coefficient, which is the dominant factor influencing the solubility of a solute. The water solubility and molecular weight play important roles in generating osmotic pressure, which means that decreasing the molecular weight and increasing the molecular polarity leads to an increase in osmotic pressure. In other words, [TBP][BS] has a higher solubility than [TBP][MBS], [TBP][DMBS], and [TBP][TMBS] because of the relatively high polarity and small size of the anionic moiety in the aqueous state. Therefore, [TBP][BS] can induce a higher osmotic pressure than [TBP][MBS], [TBP][DMBS], and [TBP][TMBS]. The osmotic pressure was found to decrease in order [TBP][BS], [TBP][MBS], [TBP][DMBS], and [TBP][TMBS], which is consistent with the order of their corresponding conductivities.

### 3.4. Thermoresponsive Behavior

**Figure 7:**
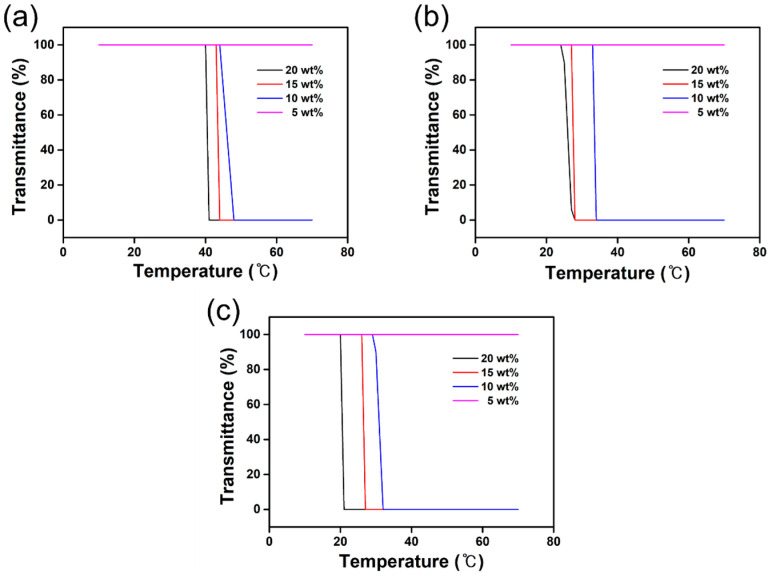
Transmittance curve of tetrabutylphosphonium (**a**) 4-methylbenzenesulfonate ([TBP][MBS]), (**b**) 2,4-dimethylbenzenesulfonate ([TBP][DMBS]), and (**c**) 2,4,6-trimethylbenzenesulfonate ([TBP][TMBS]) according to the temperature change.

To enable the reuse of the draw solution, the diluted solution must undergo a regeneration process involving separation from the water after the FO process. The LCST phenomenon can be used as a recovery method for thermoresponsive draw solutes and is the critical temperature at which the water and draw solute change from a homogeneous to a heterogeneous state on an increase in temperature. The critical temperature at which the aqueous [TBP][BS], [TBP][MBS], [TBP][DMBS], and [TBP][TMBS] solutions phase-separated was determined by measuring the transmittance of these solutions at 650 nm with respect to temperature using a UV–Vis spectrophotometer. The aqueous [TBP][BS] solution did not show any change in transmittance between 0–100 °C (data not shown), indicating that it is not thermoresponsive. However, the aqueous solutions of [TBP][MBS], [TBP][DMBS], and [TBP][TMBS] were found to have a critical temperature, as shown in Figure 7, which is crucial for recovery. When the temperatures of the aqueous [TBP][MBS], [TBP][DMBS], and [TBP][TMBS] solutions were lower than the LCST, the ion–water interactions were stronger than the ion–ion interactions between the phosphonium cation and the sulfonate anion, and, thus, [TBP][MBS], [TBP][DMBS], or [TBP][TMBS] and water formed homogeneous phases. In contrast, at temperatures above the LCST, ion–ion interactions became more dominant than the ion–water interactions, resulting in IL aggregation and the conversion of the aqueous solution to a heterogeneous phase. In addition, the LCST decreased as the concentration of the aqueous solution increased. For example, when the concentration of the aqueous solution was 10 wt%, the LCSTs of [TBP][MBS], [TBP][DMBS], and [TBP][TMBS] were 48, 33, and 30 °C, respectively, but, at 20 wt%, the LCSTs decreased to 41, 25, and 21 °C, respectively. It has been reported that an LCST near room temperature is ideal for the recovery of draw solutes, suggesting that these ILs are promising draw solutes for FO applications [70]. Therefore, the tetrabutylphosphonium benzenesulfonate-based draw solutes can be separated from water by varying the temperature, and the energy required for this could be obtained from waste heat from power plants or geothermal heat.

### 3.5. Water and Reverse Solute Fluxes

**Figure 8:**
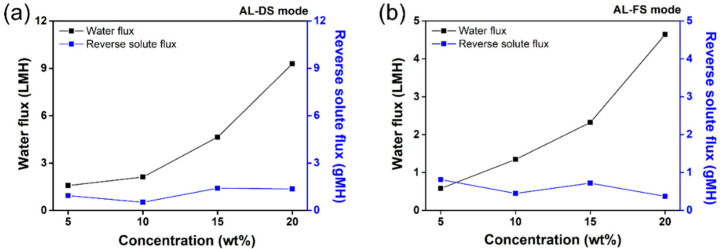
Water and reverse solute flux of tetrabutylphosphonium 2,4-dimethylbenzenesulfonate ([TBP][DMBS]) according to the concentration at room temperature in (**a**) AL-DS mode and (**b**) AL-FS mode.

To evaluate the effect of the draw solutes on FO performance, the water and reverse solute fluxes must be considered [71,72]. Based on the obtained results, which suggest good FO performance and easy recovery, [TBP][DMBS] was selected as a representative IL, and its water and reverse solute fluxes were measured at respective concentrations (5, 10, 15, and 20 wt%). The fluxes were measured during the FO process in two modes: the active layer facing the draw solution (AL-DS) mode and the active layer facing the feed solution (AL-FS) mode. Two connected glass tubes were filled with feed solution on one side and draw solution (aqueous [TBP][DMBS], 5–20 wt%) on the other side. Then, a FO membrane was placed between the connected glass tubes, and the measurements were performed at room temperature, i.e., below the LCST of the aqueous [TBP][DMBS] solution. The outcomes are shown in Figure 8. The water flux of [TBP][DMBS] improved with an increase in concentration because a higher concentration of the draw solution induces a higher osmotic pressure, thus improving the water flux in the FO system [73]. For example, in AL-DS mode, the water fluxes were approximately 1.58, 2.11, 4.64, and 9.29 LMH at concentrations of 5, 10, 15, and 20 wt%, respectively. Furthermore, in AL-FS mode, the water fluxes were approximately 0.58, 1.35, 2.32, and 4.64 LMH at concentrations of 5, 10, 15, and 20 wt%, respectively. In addition, depending on the orientation of the membrane, the water flux changes [74]. In AL-FS mode, water molecules permeate the active layer from the feed solution, thus diluting the draw solution in the porous layer. This phenomenon is known as dilution internal concentration polarization (ICP). In contrast, in AL-DS mode, the ICP effect is negligible when the feed solution is pure because it is in the porous layer [75,76]. Therefore, at all concentrations, the water flux values of aqueous [TBP][DMBS] in the AL-DS mode were larger than those in the AL-FS mode.

### 3.6. Recyclability Study of [TBP][DMBS]

**Figure 9:**
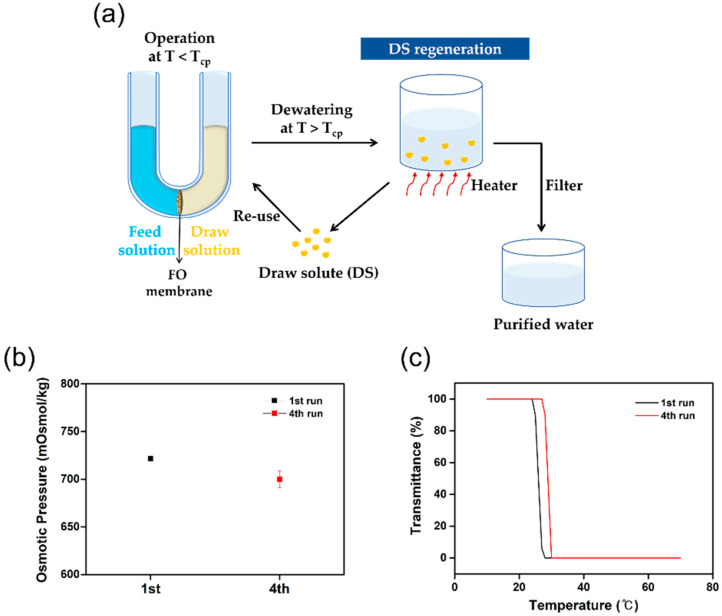
Recyclability study of [TBP][DMBS] in four cycles: (**a**) scheme of the FO system, (**b**) osmotic pressures, and (**c**) thermoresponsive behavior tests. From the 2nd to the 4th run, the recovered [TBP][DMBS] from the previous run was used.

To confirm the recyclability of [TBP][DMBS] in the water treatment field, the FO process was repeated four times using a 20 wt% solution of [TBP][DMBS] as the draw solution and DI water as the feed solution. The recycling FO system is illustrated in Figure 9a. When the temperature rises above the critical temperature after the permeation process, [TBP][DMBS] is precipitated in the solution, and pure water can be easily separated by a simple filtration process. As shown in Figure 9b,c, to confirm the recyclability of [TBP][DMBS], the osmotic pressure and thermoresponsive behavior tests of [TBP][DMBS] were measured at the 4th run, respectively. The osmotic pressure of [TBP][DMBS] at 4th run is almost the same as that of the pristine [TBP][DMBS], but the LCST value slightly increases after the 4th run. These recycling results clearly show that [TBP][DMBS] can be easily recycled with relatively low energy consumption without significant loss.

## 4. Conclusions

A series of draw solutes containing benzenesulfonate derivatives ([TBP][BS], [TBP][MBS], [TBP][DMBS], and [TBP][TMBS]) were synthesized via the anion exchange reaction of tetrabutylphosphonium bromide ([TBP][Br]), exchanging bromide for benzenesulfonate, 4-methylbenzenesulfonate, 2,4-dimethylbenzenesulfonate, and 2,4,6-trimethylbenzenesulfonate, respectively, and their suitability as draw solutes for the FO process was investigated. Aqueous [TBP][BS] was not thermoresponsive (no LCST). However, 20 wt% aqueous [TBP][MBS], [TBP][DMBS], and [TBP][TMBS] solutions were found to have LCSTs of approximately 41, 25, and 21 °C, respectively, which is useful for their recovery. Furthermore, the water and reverse solute fluxes of 20 wt% aqueous [TBP][DMBS] were measured to be approximately 9.29 LMH and 1.37 gMH, respectively, in AL-DS mode and 4.64 LMH and 0.37 gMH, respectively, in AL-FS mode. Based on the above results, tetrabutylphosphonium benzenesulfonate-based draw solutes are promising candidates for the draw solute owing to their excellent FO performance and easy recovery.

## Data Availability

The datasets are available from the corresponding author on reasonable request.
